# Obesity is not associated with poor outcomes in older patients with sepsis

**DOI:** 10.1186/cc13237

**Published:** 2014-03-17

**Authors:** MI Restrepo, P Faverio, LF Reyes, A Anzueto

**Affiliations:** 1 University of Texas Health Science Center, San Antonio, TX, USA; 2 University of Milan-Bicocca, Monza, Italy; 3 Universidad de La Sabana, Bogota, Colombia

## Introduction

Studies suggest that obesity may influence mortality in patients who develop sepsis. However, the mechanisms linked to improved outcomes are unclear. Our aim was to assess the impact of obesity on mortality at 30 and 180 days and cytokine expression.

## Methods

We used a platform of a negative randomized control trial in subjects (*n *= 51) with a diagnosis of severe sepsis with ≥1 organ failure. The cohort of severe septic subjects was stratified by obesity status based on the body mass index (BMI >30). Primary outcomes: 30-day and 180-day mortality; secondary outcome: difference in median (IQR) of five inflammatory cytokines including tumor necrosis factor alpha (TNFα), TNFα-receptor 2, interleukin (IL)-6, IL-1-receptor-antagonist (IL- 1ra) and IL-10. The measurement of median baseline cytokine levels was done in serum by Luminex technology. Statistical significance was defined as *P *< 0.05.

## Results

Fifty-one subjects with severe sepsis were included in the study; 37% of the patients were obese (BMI >30). Paradoxically, obese severe septic patients had lower 30-day mortality (*n *= 1 (5%) vs. *n *= 9 (28%), *P *= 0.069) and 180-day mortality (*n *= 1 (5%) vs. *n *= 13 (41%), *P *= 0.008), when compared with nonobese. The expression of TNFα, TNFα-receptor 2, IL-6, IL-1ra and IL-10 was not statistically significant different among obese versus nonobese severe septic patients.

## Conclusion

Obesity is associated with lower mortality rates at 30 and 180 days in patients diagnosed with severe sepsis. This survival benefit was not associated with lower cytokine production among obese patients. Further studies are needed to assess the mechanisms associated with the survival benefit related to obesity in patients with severe sepsis.

